# Ferroptosis-related genes are considered as potential targets for CPAP treatment of obstructive sleep apnea

**DOI:** 10.3389/fneur.2023.1320954

**Published:** 2023-12-21

**Authors:** Jing Huang, Hezi Zhang, Lichao Cao, Fang Chen, Weinan Lin, Qinghua Lu, Xiao Huang, Qi Weng, Qin Yang

**Affiliations:** ^1^Shantou University Medical College, Shantou, Guangdong Province, China; ^2^Shenzhen Nucleus Gene Technology Co., Ltd., Shenzhen, Guangdong Province, China; ^3^Department of Respiratory Diseases, Shenzhen Children's Hospital, Shenzhen, Guangdong Province, China; ^4^Shenzhen Pediatrics Institute of Shantou University Medical College, Shenzhen, Guangdong Province, China

**Keywords:** obstructive sleep apnea, chronic intermittent hypoxia, continuous positive airway pressure, ferroptosis, bioinformatics analysis

## Abstract

Obstructive sleep apnea (OSA) is a common syndrome characterized by upper airway dysfunction during sleep. Continuous positive airway pressure (CPAP) is the most frequently utilized non-surgical treatment for OSA. Ferroptosis play a crucial role in the physiological diseases caused by chronic intermittent hypoxia, but its involvement in the development of OSA and the exact mechanisms have incompletely elucidated. GSE75097 microarray dataset was used to identify differentially expressed genes between OSA patients and CPAP-treated OSA patients. Subsequently, Gene Ontology (GO) annotation, Kyoto Encyclopedia of Genes and Genomes (KEGG) pathway, STRING database, and FerrDb database were conducted to analyze the biological functions of differentially expressed genes and screen ferroptosis-related genes. Finally, GSE135917 dataset employed for validation. There were 1,540 differentially expressed genes between OSA patients and CPAP-treated OSA patients. These differentially expressed genes were significantly enriched in the regulation of interleukin-1-mediated signaling pathway and ferroptosis-related signaling pathway. Subsequently, 13 ferroptosis-related genes (DRD5, TSC22D3, TFAP2A, STMN1, DDIT3, MYCN, ELAVL1, JUN, DUSP1, MIB1, PSAT1, LCE2C, and MIR27A) were identified from the interaction between differentially expressed genes and FerrDb database, which are regarded as the potential targets of CPAP-treated OSA. These ferroptosis-related genes were mainly involved in cell proliferation and apoptosis and MAPK signaling pathway. Furthermore, DRD5 and TFAP2A were downregulated in OSA patients, which showed good diagnostic properties for OSA, but these abnormal signatures are not reversed with short-term effective CPAP therapy. In summary, the identification of 13 ferroptosis-related genes as potential targets for the CPAP treatment of OSA provides valuable insights into the development of novel, reliable, and accurate therapeutic options.

## 1 Introduction

Obstructive sleep apnea (OSA) is a common sleep-disordered breathing disease, occurring in all age groups from early infancy through adolescence. The primary pathological features of OSA mainly involve sleep apnea, characterized by chronic intermittent hypoxia and sleep fragmentation, which can result in oxidative damage to brain, neuronal cell injury, and chronic inflammation, contributing to the development of complications in multi-organ and systems such as coronary heart disease, hypertension, and diabetes (1–3). The main treatment for OSA is continuous positive airway pressure (CPAP), which effectively alleviates drowsiness symptoms and improves the quality of life in patients with OSA (4, 5). However, the underlying mechanism of CPAP therapy for OSA remains elusive, as the pathogenesis of OSA involves multiple biological functions, including pyroptosis, autophagy, and ferroptosis (6–8). Therefore, it is crucial to explore the potential molecular targets of CPAP therapy for OSA to further examine the pathological mechanism of OSA and improve the clinical management of patients.

Ferroptosis is a newly regulated form of cell death characterized by iron-dependent accumulation of lipid peroxidation, resulting in intracellular reactive oxygen species (ROS) and abnormal lipid metabolism, ultimately leading to cell death (9, 10). Previous studies have demonstrated the pivotal role of ferroptosis in pathological processes such as cancer, respiratory disease, cerebral hemorrhage, myocardial infarction, and ischemic stroke (11). Activation or inhibition of ferroptosis can interfere with the development of diseases, so it is of great practical significance for clinical treatment of human diseases to explore the role of ferroptosis in various diseases by sorting out ferroptosis-related genes (12). Recent studies have found that serum ferritin levels tend to rise with increasing OSA severity (13). Chronic intermittent hypoxia is the signature manifestation of OSA, and intermittent hypoxia in the brain will lead to excessive accumulation of reactive oxygen species (ROS) in neurons, which may cause permanent death of neurons (14). Recent studies have shown that hypoxia can increase the iron content in the brain of animals leading to neurodegeneration, and disorder the metabolism of nerve cells by affecting the ferroptosis-related to genes, resulting in excess oxygen free radicals (15, 16). Simultaneously, Liu et al. (17) reported that two ferroptosis-related genes can serve as biomarkers for diagnosing OSA. Abnormal iron metabolism is associated with disease severity and poor oxygenation in patients with OSA (18). However, the specific regulatory mechanism of ferroptosis in the pathophysiological process of OSA and its clinical application still need to be further studied and explored.

Here, we innovatively identified the molecular targets of CPAP-treated OSA and explored the relationship between ferroptosis and CPAP therapy in order to enhance the understanding of the transcriptomic characteristics underlying CPAP treatment of OSA.

## 2 Methods

### 2.1 Data collection and preprocessing

**Figure 1:**
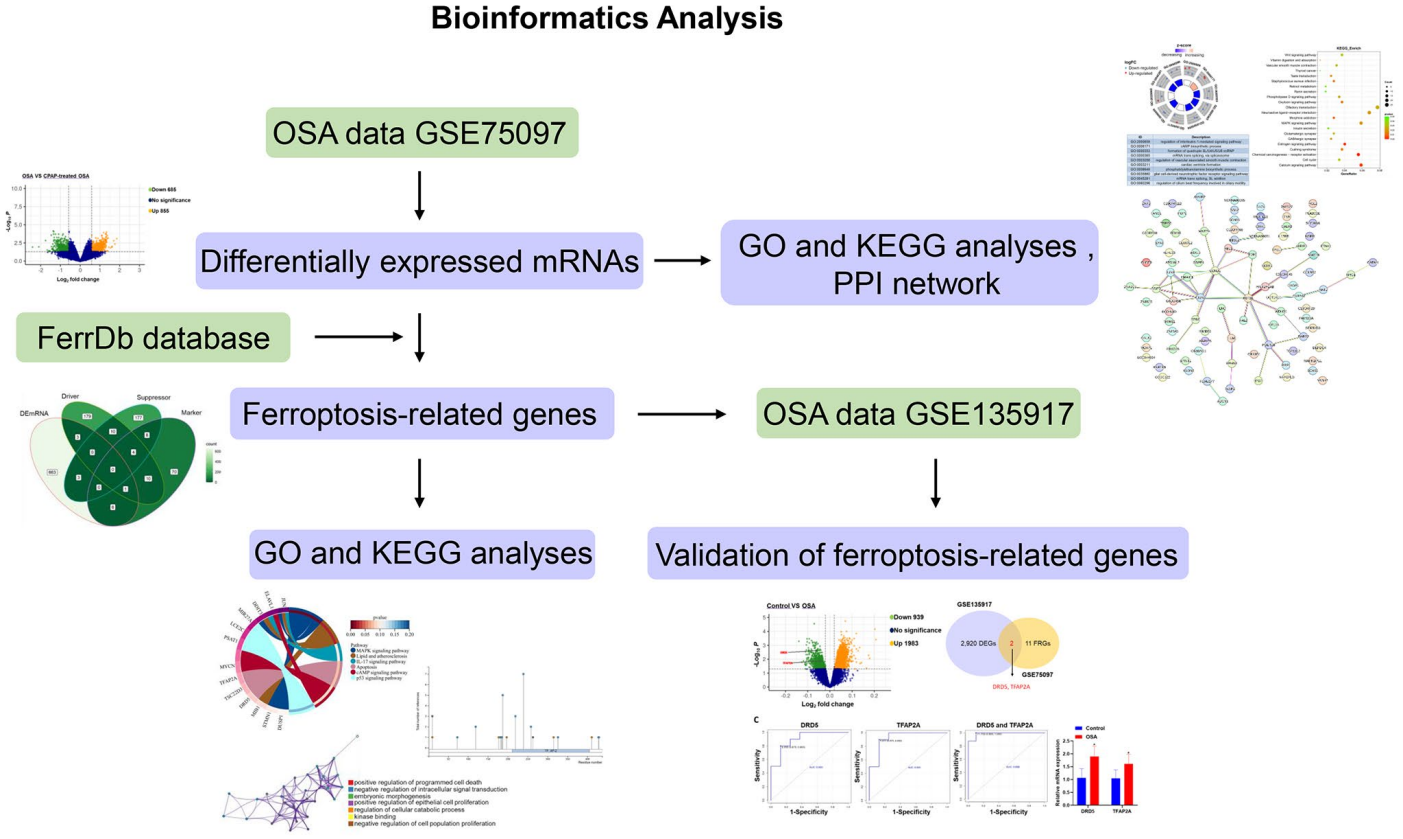
Flowchart of bioinformatics analysis.

The flow chart illustrating bioinformatics analysis is shown in Figure 1. The microarray datasets related to OSA (GSE75097 and GSE135917) were obtained from the Gene Expression Omnibus (GEO) database^1^ (19, 20). As indicated in Table 1, the sequencing platform of GSE75097 is GPL10904, and GSE135917 is GPL6244. There are 28 OSA patients and 14 OSA patients undergoing CPAP treatment in GSE75097. Additionally, GSE135917 included 8 health subjects and 10 OSA patients, as well as 24 patients with OSA at baseline and 24 after exposure to CPAP. Peripheral blood mononuclear cells (PBMC) samples from GSE75097 were included for screening differentially expressed mRNAs (DEmRNAs) and ferroptosis-related genes. Meanwhile, GSE135917 was employed to validate the diagnostic value of ferroptosis-related genes. The raw data in cell format files were read using the “affy” package, followed by normalization of the raw data using the Affymetrix platform in the R package and data processing with RMA functions.

**Table 1:** Detailed information of GSE75097 and GSE135917 datasets.

Accession	Platform	Sample	Normal	OSA	CPAP-OSA	Gene	Application
GSE75097	GPL10904	PBMC	0	28	14 (1 year)	mRNA	Training
GSE135917	GPL6244	Fat Tissue	8	10/24 (OSA at baseline)	24 (2 weeks)	mRNA	Verification

OSA, Obstructive sleep apnea; CPAP, Continuous positive airway pressure; the design of GSE135917: (1) 10 subjects with OSA vs. 8 controls; (2) 24 subjects with OSA at baseline vs. after effective CPAP therapy.

### 2.2 Screening of differentially expressed genes

The limma package was used to identify DEmRNAs, considering genes with a *p* value < 0.05 and |log_2_-fold change (FC)| > 1.5 as significant. Volcano plots generated using the “ggplot2” packages (version 3.3.3) of R software (version 3.6.3) were employed for visualizing the identified DEmRNAs.

### 2.3 Functional enrichment analysis

The Gene Ontology (GO) annotation and Kyoto Encyclopedia of Genes and Genomes (KEGG) pathway enrichment analyses of DEmRNAs were conducted using DAVID 6.8 (21) and Metascape^2^ (22) *p* < 0.05 was applied for statistical analysis.

### 2.4 Protein–protein interaction network creation

Based on the online STRING database,^3^ the interactions of DEmRNAs were predicted by constructing and analyzing PPI networks. In the PPI network, target genes are represented as nodes, while the lines between nodes depict related interactions. The color of these lines reflects the intensity of the interactions.

### 2.5 Identification of ferroptosis-related genes

We obtained ferroptosis-related genes from the FerrDb database,^4^ encompassing “Driver,” “Suppressor,” and “Marker.” Subsequently, Venn diagram was used to demonstrate the overlap between DEmRNAs and ferroptosis-related genes in the FerrDb database. GO and KEGG pathway analyses were conducted using the Metascape website and DAVID 6.8. PhosphoSitePlus^5^ was performed to examine protein translational modifications (PTMs) of ferroptosis-related genes.

### 2.6 Validation of the identified ferroptosis-related genes

HiPlot software was applied to perform the receiver operating characteristic (ROC) curve analysis. SPSS 22.0 version was used for binary logistic regression to calculate the prediction probability of multiple genes in each sample, enabling multigene ROC analysis. The area under the ROC curve (AUC) was adopted as a quantitative measure to evaluate the results, with genes exhibiting an AUC > 0.8 considered to have exceptional diagnostic performance.

### 2.7 Quantitative real−time PCR

Three healthy subjects and three OSA patients were recruited from October 2023 to November 2023 at Shenzhen Children’s Hospital Affiliated to Shantou University Medical College. The study was approved by the Ethics Committee of Shenzhen Children’s Hospital Affiliated to Shantou University Medical College (202309102). Peripheral blood samples were collected for q-PCR detection. Total RNA was extracted from blood using TRNzol Universal Total RNA extraction reagent (TIANGEN, Beijing, China) according to manufacturer’s instructions. The cDNA was synthesized using Hifair® III 1st Strand cDNA Synthesis SuperMix (YESEN, Shanghai, China). On an ABI Stepone plus PCR equipment, q-PCR was performed using Hieff® qPCR SYBR Green Master Mix (YESEN, Shanghai, China). The following were the primers utilized in this study: DRD5-Forward: GGGCAGTTCGCTCTATACCAG; DRD5-Reverse: GGTCCAGATGATGAGTAGGGTC; TFAP2A-Forward: AGGTCAATCTCCCTACACGAG; TFAP2A-Reverse: GGAGTAAGGATCTTGCGACTGG; GAPDH-Forward: TGGAAATCCCATCACCATCT; and GAPDH-Reverse: TGGACTCCACGACGTACTCA. Relative quantification was determined using the 2^–∆∆Ct^ method.

### 2.8 Statistical analysis

Data were expressed as mean ± standard deviation (SD) and the Graph Pad 8.0 were performed for analysis in this study. The Student’s *t*-test was utilized to determine the difference between the two groups. *p* < 0.05 was considered significant.

## 3 Results

### 3.1 Identification of DEmRNAs between OSA and CPAP-treated OSA

**Figure 2:**
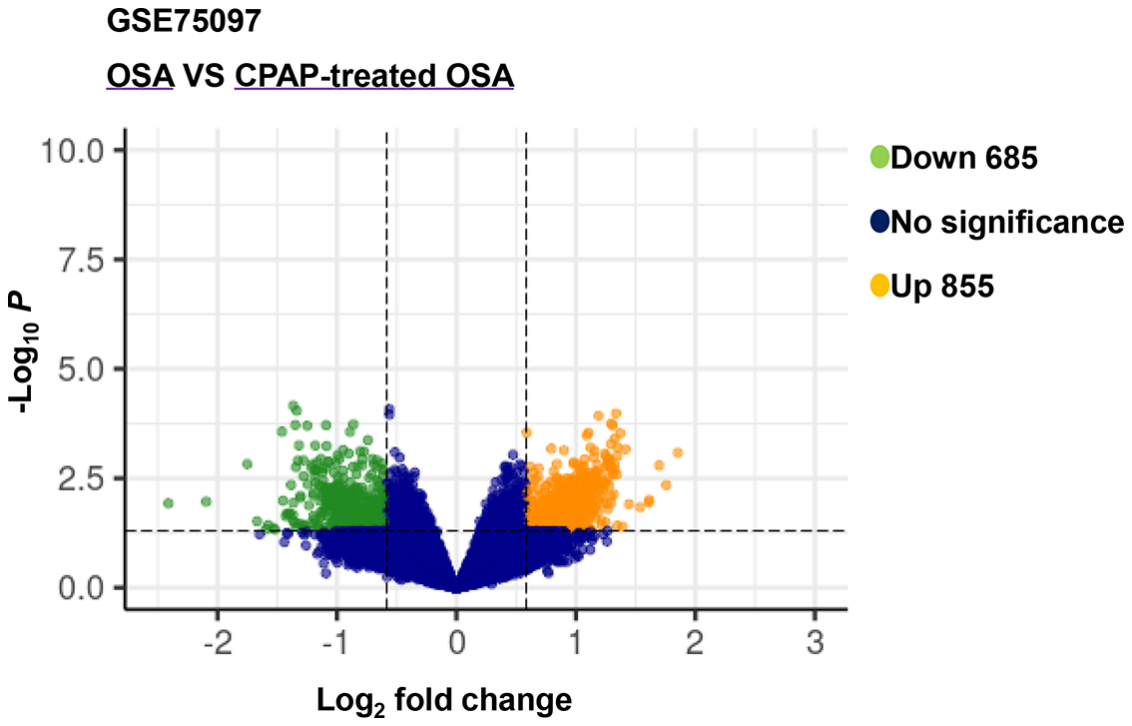
Volcano plot of differentially expressed genes between OSA and CPAP-treated OSA in GSE75097.

After analyzing the expression profiling data from GSE75097, a total of 1,540 DEmRNAs, including 685 downregulated and 855 upregulated genes, were selected from OSA and CPAP-treated OSA by applying screening criteria of |log_2_(FC)| > 1.5 and value of *p* < 0.05 (Figure 2). Additionally, Table 2 presented the top five upregulated genes and the top five downregulated genes, which included LOC401131, LOC643324, TNR, LOC645726, HIRIP3, RNU4ATAC, OXGR1, JUNB, LOC646513, and RNU11.

**Table 2:** The top five of upregulated genes and downregulated genes.

Gene	logFC	*p* value	Up/Down
LOC401131	1.336715442	0.000105821	Up
LOC643324	1.189998627	0.0001183	Up
TNR	1.297333582	0.00017904	Up
LOC645726	1.307407514	0.000198165	Up
HIRIP3	0.587443645	0.000285797	Up
RNU4ATAC	−1.366824666	0.000069506	Down
OXGR1	−1.336719153	0.000090044	Down
JUNB	−0.862500468	0.000185445	Down
LOC646513	−1.092160889	0.000192185	Down
RNU11	−1.348415132	0.000192794	Down

### 3.2 GO and KEGG enrichment analysis for DEmRNAs

**Figure 3:**
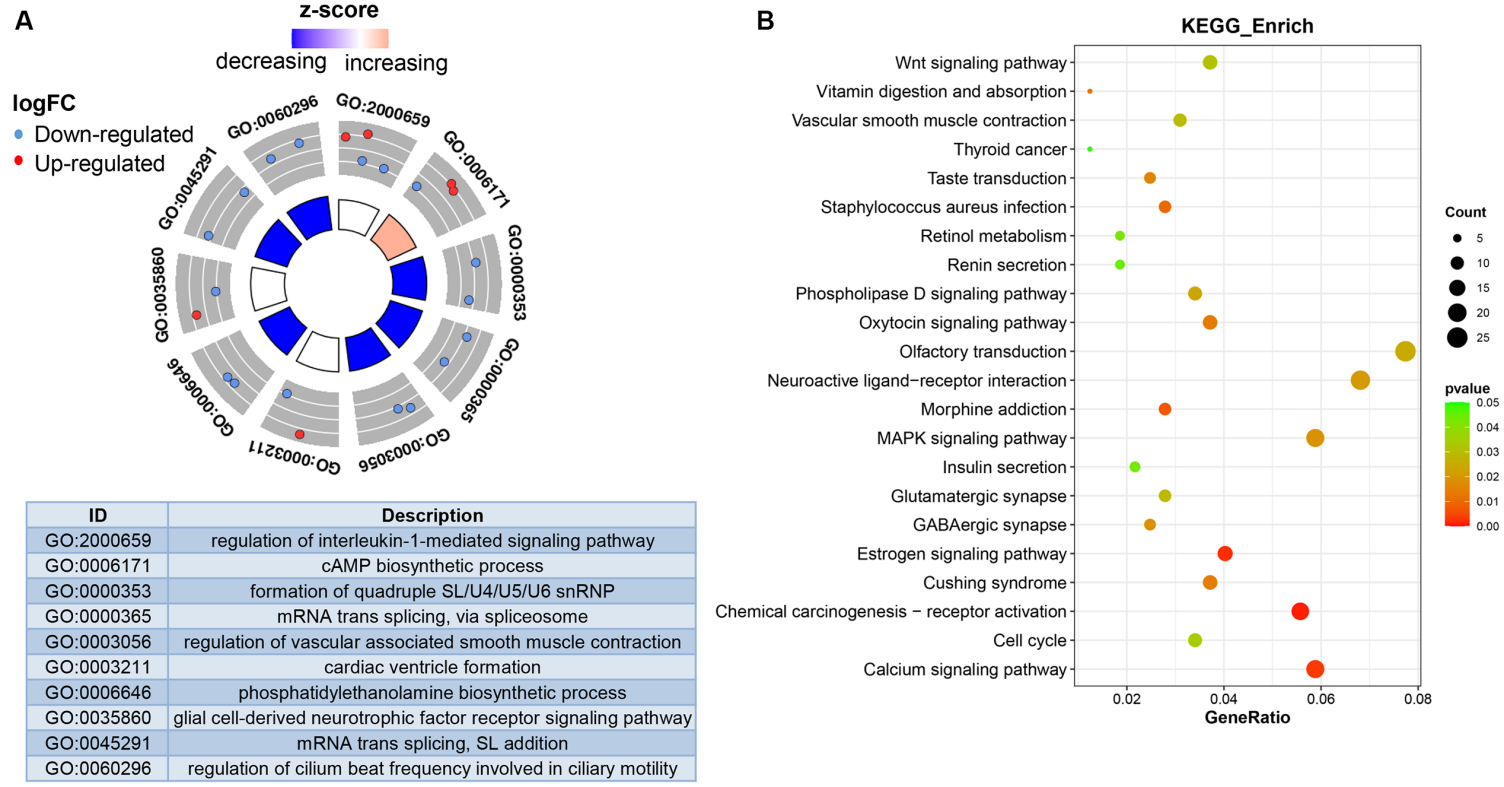
Functional analysis of differentially expressed genes. **(A)** Differentially expressed genes-enriched GO categories. **(B)** KEGG pathways analysis of differentially expressed genes.

To further understand the biological function of DEmRNAs, we conducted functional enrichment analysis. The results of GO terms enrichment analysis indicated that the genes significantly enriched were involved in “regulation of interleukin-1-mediated signaling pathway,” “cAMP biosynthetic process,” “regulation of vascular associated smooth muscle contraction,” and “phosphatidylethanolamine biosynthetic process” (Figure 3A). KEGG pathways revealed that DEmRNAs mainly involved the “Wnt signaling pathway,” “MAPK signaling pathway,” “Calcium signaling pathway,” and “Vascular smooth muscle contraction” (Figure 3B). Therefore, it can be reasonably speculated that biological functions mediated by DEmRNAs are involved in the physiological processes of OSA treated with CPAP.

### 3.3 PPI network analysis of DEmRNAs

**Figure 4:**
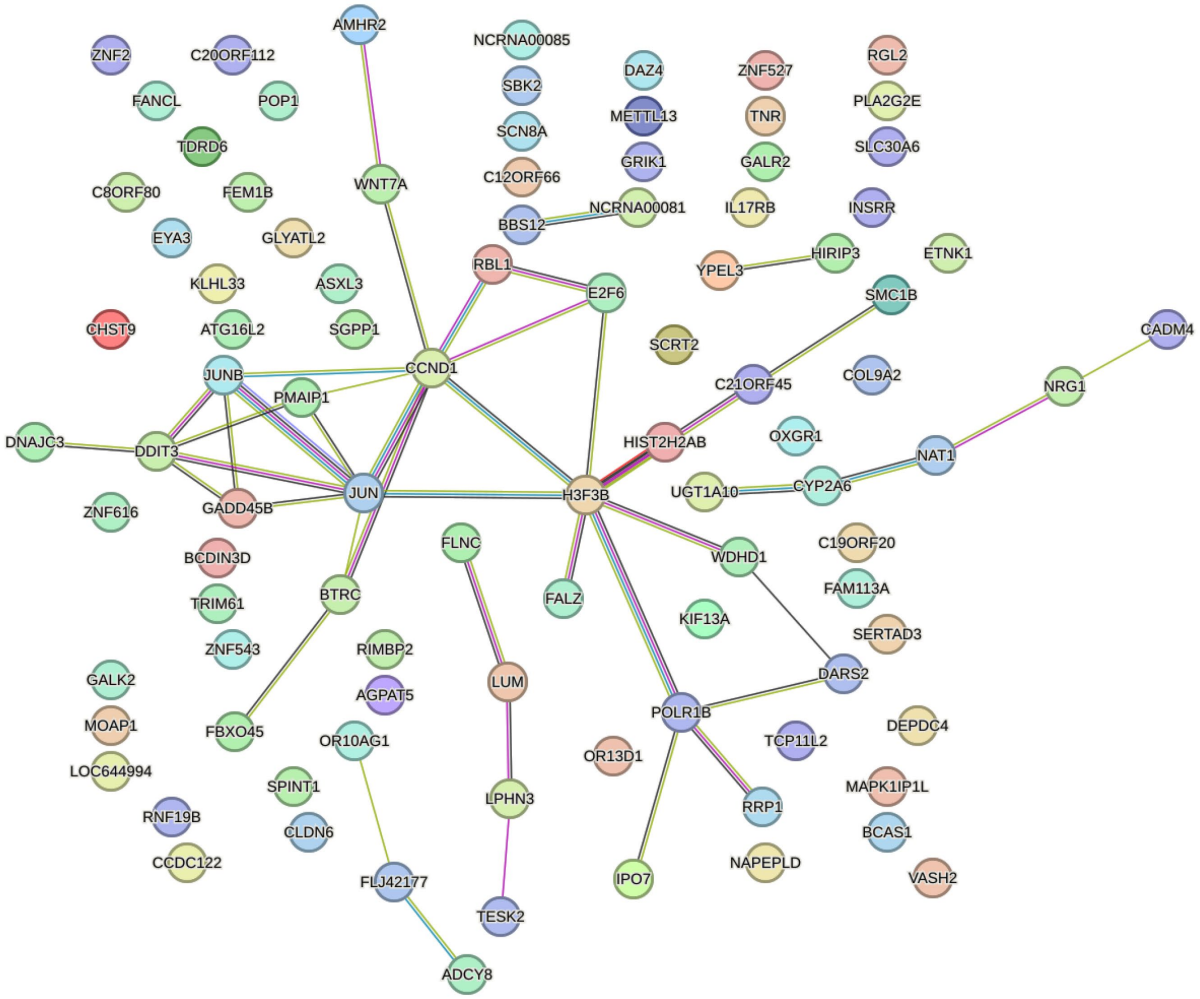
STRING database was used to construct PPI network.

The PPI network demonstrated the interaction between DEmRNAs. We constructed the PPI network to distinguish the connections among the top 200 DEmRNAs above. As depicted in Figure 4, the network consisted of 96 nodes and 44 edges, with an average local clustering coefficient is 0.244. The PPI network diagram showed JUN, CCDN1, and H3-3B were the most interacting proteins.

### 3.4 Identification of ferroptosis-related genes, biological function analysis, and PTMs sites prediction

**Figure 5:**
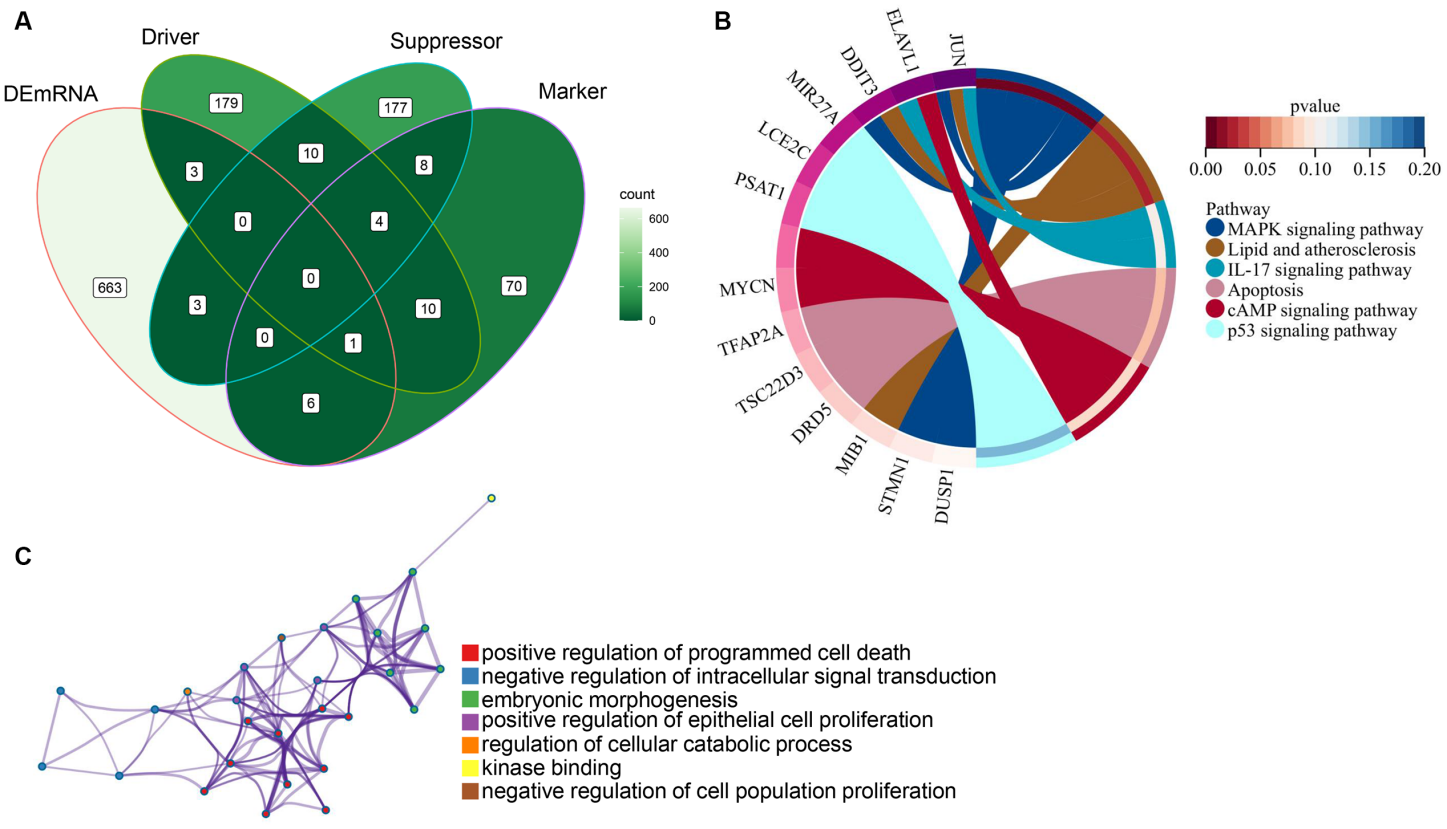
Identification and functional enrichment analysis of ferroptosis-related genes. **(A)** Ferroptosis-related genes were obtained from the intersection of the FerrDb database with differentially expressed genes. **(B)** KEGG pathways analysis of ferroptosis-related genes using the Metascape website and DAVID database. **(C)** GO categories of ferroptosis-related genes using the Metascape website.

Our study found that DEmRNAs are significantly enriched in the WNT signaling pathway. Some studies have reported activation of Wnt/beta-catenin signaling attenuated intracellular lipid ROS production, thereby inhibiting ferroptosis in gastric cancer cells (23). To investigate the association between DEmRNAs and ferroptosis, we further screened differentially ferroptosis-related genes according to the FerrDb database and DEmRNAs. Venn diagram analysis of the FerrDb database (included “Drivers,” “Suppressors,” and “Makers”) and DEmRNAs identified the following genes which were differentially expressed in ferroptosis: DRD5, TSC22D3, TFAP2A, STMN1, DDIT3, MYCN, ELAVL1, JUN, DUSP1, MIB1, PSAT1, LCE2C, and MIR27A (Figure 5A; Table 3). Subsequently, these ferroptosis-related genes were uploaded to Metascape website and DAVID database, and it was shown that the biological pathways were remarkably enriched involvement of ferroptosis-associated pathways, such as MAPK signaling pathway, Lipid and atherosclerosis pathway, and IL-17 signaling pathway (Figure 5B). Additionally, GO analysis indicated that these ferroptosis-related genes regulate cell proliferation and death (Figure 5C). Besides, we predicted the PTMs sites of genes associated with ferroptosis. Except for DRD5, LEC2C, and MIR27A, PTMs were found in all genes (Supplementary Figures S1, S2), which is crucial for us to understand cellular signaling pathways. Taken together, we screened 13 ferroptosis-related genes that are considered as therapeutic targets for CPAP-treated OSA.

**Table 3:** Details of 13 ferroptosis-related genes.

Gene	logFC	*p* value	Up/Down
DDIT3	−0.7886347	0.001624304	Down
DRD5	0.7604257	0.034925645	Up
DUSP1	−0.6341032	0.013720922	Down
ELAVL1	0.9008274	0.021881973	Up
JUN	−1.2806116	0.002822219	Down
LCE2C	0.8773638	0.016244034	Up
MIB1	0.6993263	0.031003364	Up
MIR27A	0.8125076	0.010206382	Up
MYCN	−0.6484463	0.044747900	Down
PSAT1	1.0379086	0.011651924	Up
STMN1	0.6377915	0.015467237	Up
TFAP2A	1.1186158	0.046086407	Up
TSC22D3	−0.6130752	0.015658925	Down

### 3.5 DRD5 and TFAP2A were key genes in the pathogenesis of OSA

**Figure 6:**
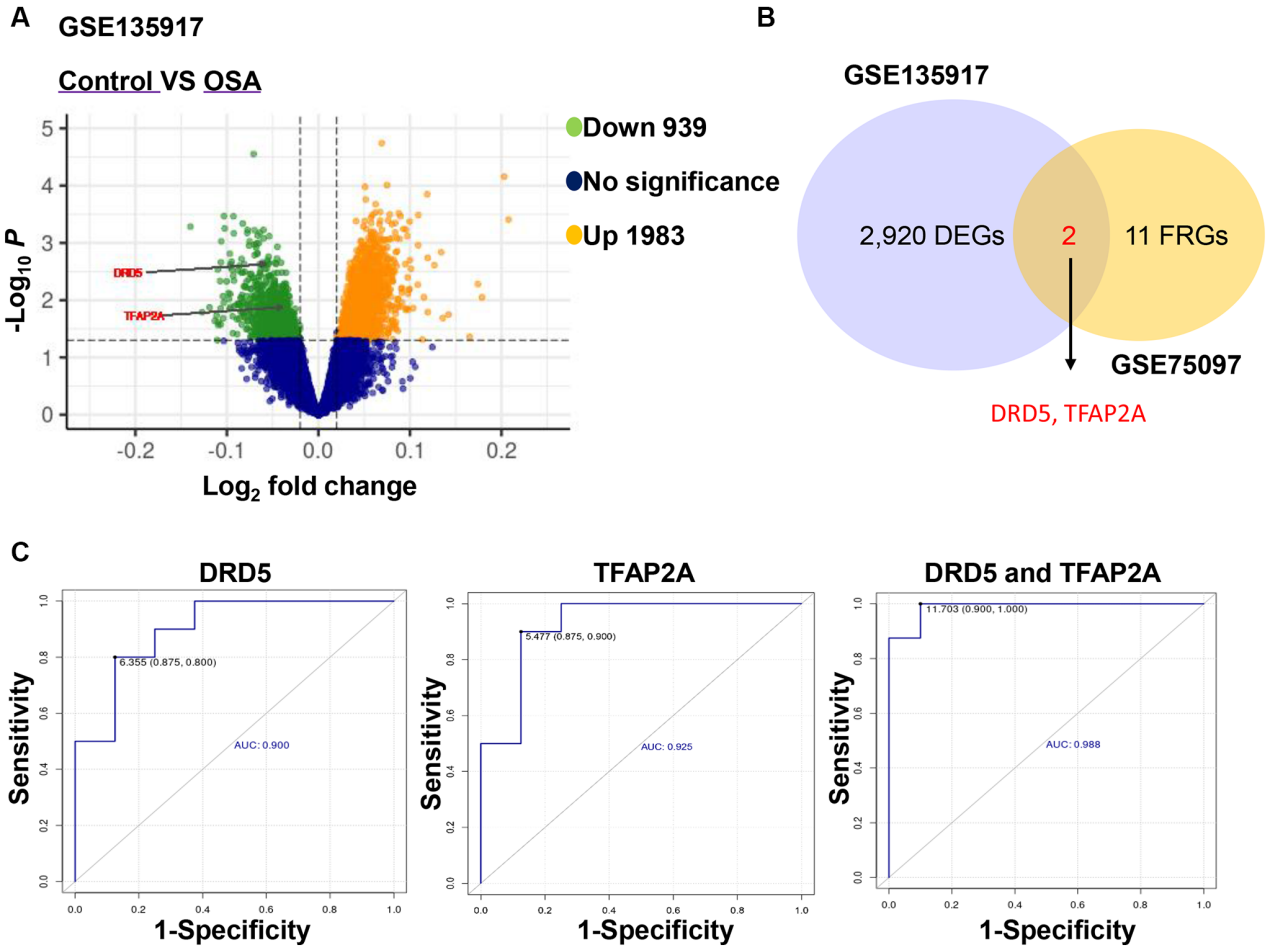
DRD5 and TFAP2A had the diagnostic value for OSA. **(A)** Volcano plot of differentially expressed genes between healthy samples and OSA in GSE135917. **(B)** Venn diagram showed the overlapping genes of 13 ferroptosis-related genes and differentially expressed genes of GSE135917. DEGs indicates differentially expressed genes and FRGs ferroptosis-related genes. **(C)** The diagnostic ROC curves of DRD5 and TFAP2A in OSA and healthy samples.

Obesity is a significant risk factor for developing OSA patients, and the interaction between the respiratory system and adipose tissue plays a crucial role in the preventing and treatment of OSA (24, 25). Compared to control patients, OSA patients exhibited 939 downregulated genes and 1,983 upregulated genes (Figure 6A). Interestingly, two of these genes overlapped with 13 ferroptosis-related genes obtained as described above, namely, DRD5 and TFAP2A (Figure 6B). Therefore, we speculated that DRD5 and TFAP2A are not only potential targets for CPAP treatment of OSA, but also key genes in OSA pathogenesis. Subsequently, ROC curves were generated to assess the diagnostic value of DRD5 and TFAP2A. The results demonstrated that both DRD5 (AUC = 0.900) and TFAP2A (AUC = 0.925), as well as their combination (AUC = 0.988), held significant potential for diagnosing OSA patients (Figure 6C). GSE75097 revealed an increase in the expressions of DRD5 and TFAP2A in PBMC samples from CPAP-treated OSA patients compared to those with OSA (Table 3). However, CPAP therapy did not improve the abnormal expression of DRD5 and TFAP2A in adipose tissue among OSA patients in the GSE135917 dataset (Supplementary Table S1). We thought that this may be due to the long duration of CPAP treatment (1 year) in GSE75097 (19), as opposed to 2 weeks in GSE135917 (20), which could account for these abnormal expression of DRD5 and TFAP2A was not improved with effective but short-term CPAP therapy.

In addition, in order to further explore the clinical expression of DRD5 and TFAP2A, we collected peripheral blood samples from healthy subjects and OSA patients for examination (due to limited clinical resources, we do not obtain the OSA subject before CPAP and OSA patient with long-term CPAP treatment). Compared with healthy subjects, the expression of DRD5 and TFAP2A in OSA patients was significantly upregulated (Supplementary Figure S3, ^*^p < 0.05), which was contrary to the results in Figure 6A. We analyzed the reasons for this: (1) there were too few clinical samples, so more clinical samples needed to be included to verify the reliability of the results; (2) Since the risk factors of OSA pathogenesis is related to age, gender, and obesity (26), it is necessary to consider these clinical factors in the included samples to ensure the correctness of the results. Therefore, we will include more clinical trials for verification in the future, and explore the potential molecular mechanism *in vivo* and *in vitro* experiments.

Collectively, these results indicated that DRD5 and TFAP2A, as potential targets of CPAP for OSA treatment, were also key genes in the pathogenesis of OSA, and had good diagnostic value. Nevertheless, due to the complex relationship between the pathogenesis of OSA and age, gender, and obesity, and the relationship between OSA treatment and these risk factors is still unclear, we still need to further explore the deeper molecular mechanism through *in vitro* and *in vivo* experiments, and include more detailed clinical samples to verify from different perspectives.

## 4 Discussion

Continuous positive airway pressure is the primary treatment for OSA, but due to limited data on molecular and pathological mechanisms, the interaction of target genes induced by CPAP therapy in OSA patients is unknown. The latest research confirms that serum ferritin levels tend to rise with increasing OSA severity (13). Besides, chronic intermittent hypoxia is the main pathophysiological mechanism of OSA. Since chronic intermittent hypoxia can trigger an increase in intracellular ROS, it may cause cellular ferroptosis (27). Previous studies have shown that ferroptosis plays an important role in chronic intermittent hypoxia-induced liver and heart damage (8, 28). Here, we screened 13 ferroptosis-related genes as treatment targets for CPAP-treated OSA, and the ROC of combined DRD5 and TFAP2A was 0.988, which had the diagnostic value for OSA.

Currently, 2–4% of adults suffer from OSA (29), and its prevalence has increased with the obesity epidemic (30). Clinically, CPAP is a common means of effective treatment of OSA, improving clinical symptoms in patients with OSA, such as excessive sleepiness and improved sleep therapy (31, 32), CPAP can also reduce cardiovascular risk in moderate-to-severe OSA patients by reducing circulating markers of cardiovascular risk (33, 34) and blood pressure (35). However, how CPAP improves OSA at the molecular level remains unknown. In our study, CPAP-treated OSA patients had 1,540 differentially expressed genes in their blood compared to OSA patients, with 685 downregulated and 855 upregulated genes, indicating CPAP treated OSA patients by regulating transcriptional signatures. Moreover, these DEmRNAs were mainly enriched in “Wnt signaling pathway,” “MAPK signaling pathway,” and “Calcium signaling pathway.” Abnormal Wnt/β-catenin signaling pathway caused by chronic intermittent hypoxia, which is the most typical pathophysiological component of OSA (36). Meanwhile, Wang et al. (37) demonstrated that chronic intermittent hypoxia-mediated MAPK signaling pathway disturbed insulin secretion, which may be of important meaning for the clinical treatment of OSA. Calcium (Ca^2+^) and ROS are multifunctional signaling molecules that coordinate physiological and pathophysiological processes (38), thus maintaining Ca^2+^ and ROS homeostasis may be critical for the clinical treatment of OSA. Therefore, these pathways may contain potential markers and future drug intervention targets of OSA.

Chronic intermittent hypoxia is the main clinical feature of OSA patients. Chen et al. (39) reported chronic intermittent hypoxia induced intracellular ROS accumulation, which leads to ferroptosis. Simultaneously, DEmRNAs-enriched signaling pathways (such as Wnt signaling pathway, MAPK signaling pathway, and Calcium signaling pathway) are also involved in the regulation of ferroptosis (23, 40, 41), so we speculated that ferroptosis-related genes are the molecular targets of CPAP treatment of OSA. Finally, we screened 13 ferroptosis-related genes (DRD5, TSC22D3, TFAP2A, STMN1, DDIT3, MYCN, ELAVL1, JUN, DUSP1, MIB1, PSAT1, LCE2C, and MIR27A) as potential targets for improving OSA with CPAP. DRD5 is a biomarker associated with ferroptosis that can be used for disease diagnosis and treatment monitoring in breast cancer (42). In addition, the production of mitochondrial ROS was increased in the renal cortex of DRD5 knockout mice (43). Furthermore, TFAP2A was upregulated in gallbladder carcinoma, promoting the increase of Fe^2+^ and malondialdehyde levels, and TFAP2A silencing attenuated the expression of key genes associated with oxidative stress (44). Our study found that DRD5 and TFAP2A expressions were upregulated in OSA patients after CPAP treatment, suggesting that CPAP may improve abnormal ROS levels and inhibited chronic intermittent hypoxia-caused ferroptosis caused by targeting elevating the expressions of DRD5 and TFAP2A. In addition, DUSP1 is a hub-gene shared of OSA and Alzheimer Disease (AD) patients, and DUSP1-mediated oxidative stress involved in the pathogenesis of OSA affecting AD (45). DUSP1 gene expression was inhibited after CPAP treatment for OSA (46), which is similar to our findings. Besides, DDIT3 has been shown to be a diagnostic target for patients with AD (47). Considering that OSA-mediated chronic intermittent hypoxia affected the pathogenesis of AD, DDIT3 may also be a key gene in ameliorating OSA-associated complications. These results were helpful for the diagnosis and treatment of OSA, and may lay the foundation for further investigation of the molecular mechanism of CPAP treatment of OSA.

## 5 Conclusion

In summary, we identified 13 ferroptosis-related genes (DRD5, TSC22D3, TFAP2A, STMN1, DDIT3, MYCN, ELAVL1, JUN, DUSP1, MIB1, PSAT1, LCE2C, and MIR27A) as the target genes induced by CPAP therapy (1 year) in the patient with OSA. Moreover, DRD5 and TFAP2A were key genes in the pathogenesis of OSA, which showed good diagnostic properties for OSA, but these abnormal expression are not reversed with short-term effective CPAP therapy (2 weeks). These results provided a reference for further research on the therapeutic mechanism and develop on drug intervention targets of OSA.

## 6 Limitations

There are some limitations to our study. The sample size of our screened dataset is relatively small, and our discoveries need to be validated in a larger sample. Second, we demonstrated that ferroptosis-related genes (DRD5 and TFAP2A) exhibited good diagnostic properties in OSA patients. However, considering that the CPAP intervention group in the GSE135917 dataset may not be sufficient to completely eliminate OSA-related abnormal transcriptional signatures due to the short duration of CPAP treatment. Besides, OSA pathogenesis-related risk factors such as age, gender, and obesity should be considered in the included clinical samples to better explain the representative genes of OSA pathogenesis and explore effective targets for CPAP therapy. Therefore, based on these limitations, we will continue to explore the molecular mechanism of CPAP therapy for OSA *in vivo* and *in vitro* experiments.

## Data availability statement

The datasets presented in this study can be found in online repositories. The names of the repository/repositories and accession number(s) can be found in the article/Supplementary material.

## Ethics statement

The studies involving humans were approved by the Ethics Committee of Shenzhen Children’s Hospital Affiliated to Shantou University Medical College (202309102). The studies were conducted in accordance with the local legislation and institutional requirements. Written informed consent for participation in this study was provided by the participants’ legal guardians/next of kin.

## Author contributions

JH: Conceptualization, Writing – original draft. HZ: Conceptualization, Writing – original draft. LC: Investigation, Software, Writing – review & editing. FC: Methodology, Writing – review & editing. WL: Data curation, Writing – review & editing. QL: Data curation, Writing – review & editing. XH: Data curation, Writing – review & editing. QW: Methodology, Writing – review & editing. QY: Conceptualization, Funding acquisition, Project administration, Writing – review & editing.

## Footnotes

^1^https://www.ncbi.nlm.nih.gov/geo/

^2^http://metascape.org

^3^https://string-db.org/

^4^http://www.zhounan.org/ferrdb/

^5^https://www.phosphosite.org/homeAction

## Supplementary material

SUPPLEMENTARY FIGURE S1 PhosphoSitePlus lollipop plots of 6 ferroptosis-related genes. Circles 362 indicate PTM sites with a height reflecting the number of references describing the 363 site.

SUPPLEMENTARY FIGURE S2 PhosphoSitePlus lollipop plots of 4 ferroptosis-related genes. Circles indicate PTM sites with a height reflecting the number of references describing the site.

SUPPLEMENTARY FIGURE S3 Clinical validation of DRD5 and TFAP2A expression (**P* < 0.05, *n* = 3).

## References

[1] Lee MH Sin S Lee S Wagshul ME Zimmerman ME. Cortical thickness and hippocampal volume in adolescent children with obstructive sleep apnea. Sleep. (2023) 46:1–11. doi: 10.1093/sleep/zsac201, PMID: 36006869 PMC9995789

[2] Yeghiazarians Y Jneid H Tietjens JR Redline S Brown DL El-Sherif N. Obstructive sleep apnea and cardiovascular disease: a scientific statement from the American Heart Association. Circulation. (2021) 144:e56–67. doi: 10.1161/cir.0000000000000988, PMID: 34148375

[3] Reutrakul S Mokhlesi B. Obstructive sleep apnea and diabetes: a state of the art review. Chest. (2017) 152:1070–86. doi: 10.1016/j.chest.2017.05.009, PMID: 28527878 PMC5812754

[4] Antic NA Catcheside P Buchan C Hensley M Naughton MT Rowland S. The effect of CPAP in normalizing daytime sleepiness, quality of life, and neurocognitive function in patients with moderate to severe OSA. Sleep. (2011) 34:111–9. doi: 10.1093/sleep/34.1.111, PMID: 21203366 PMC3001789

[5] Nokes B Cooper J Cao M. Obstructive sleep apnea: personalizing CPAP alternative therapies to individual physiology. Expert Rev Respir Med. (2022) 16:917–29. doi: 10.1080/17476348.2022.2112669 35949101

[6] Yu LM Zhang WH Han XX Li YY Lu Y Pan J. Hypoxia-induced ROS contribute to myoblast Pyroptosis during obstructive sleep apnea via the NF-κB/HIF-1α signaling pathway. Oxidative Med Cell Longev. (2019) 2019:4596368. doi: 10.1155/2019/4596368, PMID: 31885794 PMC6927050

[7] Ding H Guo H Cao J. The importance of autophagy regulation in obstructive sleep apnea. Sleep Breath. (2021) 25:1211–8. doi: 10.1007/s11325-020-02261-4, PMID: 33394324

[8] Huang J Xie H Yang Y Chen L Lin T Wang B. The role of ferroptosis and endoplasmic reticulum stress in intermittent hypoxia-induced myocardial injury. Sleep Breath. (2023) 27:1005–11. doi: 10.1007/s11325-022-02692-1, PMID: 35951213

[9] Dixon SJ Lemberg KM Lamprecht MR Skouta R Zaitsev EM Gleason CE. Ferroptosis: an iron-dependent form of nonapoptotic cell death. Cells. (2012) 149:1060–72. doi: 10.1016/j.cell.2012.03.042 PMC3367386 22632970

[10] Park E Chung SW. ROS-mediated autophagy increases intracellular iron levels and ferroptosis by ferritin and transferrin receptor regulation. Cell Death Dis. (2019) 10:822. doi: 10.1038/s41419-019-2064-5 31659150 PMC6817894

[11] Zhang JJ Du J Kong N Zhang GY Liu MZ Liu C. Mechanisms and pharmacological applications of ferroptosis: a narrative review. Ann Transl Med. (2021) 9:1503. doi: 10.21037/atm-21-1595, PMID: 34805365 PMC8573439

[12] He P Xu S Miao Z Que Y Chen Y Li S. Anti-Her2 affibody-decorated arsenene nanosheets induce ferroptosis through depleting intracellular GSH to overcome cisplatin resistance. J Nanobiotechnol. (2023) 21:203. doi: 10.1186/s12951-023-01963-7, PMID: 37370105 PMC10294336

[13] Seifen C Pordzik J Huppertz T. Serum ferritin levels in severe obstructive sleep apnea. Diagnostics. (2023) 13:1154. doi: 10.3390/diagnostics13061154, PMID: 36980461 PMC10047524

[14] Zhao H Lin J Sieck G Haddad GG. Neuroprotective role of Akt in hypoxia adaptation in Andeans. Front Neurosci. (2020) 14:607711. doi: 10.3389/fnins.2020.607711 33519361 PMC7843528

[15] Stockwell BR Friedmann Angeli JP Bayir H Bush AI Conrad M Dixon SJ. Ferroptosis: a regulated cell death Nexus linking metabolism, redox biology, and disease. Cells. (2017) 171:273–85. doi: 10.1016/j.cell.2017.09.021, PMID: 28985560 PMC5685180

[16] Lin CH Wu JS Hsieh PC. Wild bitter melon extract abrogates hypoxia-induced cell death via the regulation of ferroptosis, ER stress, and apoptosis in microglial BV2 cells. Evid Based Complement Alternat Med. (2022) 2022:1072600–8. doi: 10.1155/2022/1072600, PMID: 35449822 PMC9017512

[17] Liu P Zhao D Pan Z Tang W Chen H Hu K. Identification and validation of ferroptosis-related hub genes in obstructive sleep apnea syndrome. Front Neurol. (2023) 14:1130378. doi: 10.3389/fneur.2023.1130378, PMID: 36937508 PMC10018165

[18] Marchi NA Pizzarotti B Solelhac G. Abnormal brain iron accumulation in obstructive sleep apnea: a quantitative MRI study in the Hypno Laus cohort. J Sleep Res. (2022) 31:e13698. doi: 10.1111/jsr.13698, PMID: 35830960 PMC9787990

[19] Chen YC Chen KD Su MC Chin CH Chen CJ Liou CW. Genome-wide gene expression array identifies novel genes related to disease severity and excessive daytime sleepiness in patients with obstructive sleep apnea. PLoS One. (2017) 12:e0176575. doi: 10.1371/journal.pone.0176575, PMID: 28520763 PMC5435176

[20] Gharib SA Hurley AL Rosen MJ Spilsbury JC Schell AE Mehra R. Obstructive sleep apnea and CPAP therapy alter distinct transcriptional programs in subcutaneous fat tissue. Sleep. (2020) 43:1–11. doi: 10.1093/sleep/zsz314, PMID: 31872261 PMC7294406

[21] DennisGJrShermanBTHosackDAYangJGaoWLaneHC. DAVID: database for annotation, visualization, and integrated discovery. Genome Biol. (2003) 4:P3. doi: 10.1186/gb-2003-4-5-p312734009

[22] Zhou Y Zhou B Pache L Chang M. Metascape provides a biologist-oriented resource for the analysis of systems-level datasets. Nat Commun. (2019) 10:1523. doi: 10.1038/s41467-019-09234-6, PMID: 30944313 PMC6447622

[23] Wang Y Zheng L Shang W Yang Z Li T Liu F. Wnt/beta-catenin signaling confers ferroptosis resistance by targeting GPX4 in gastric cancer. Cell Death Differ. (2022) 29:2190–202. doi: 10.1038/s41418-022-01008-w 35534546 PMC9613693

[24] Bonsignore MR. Obesity and obstructive sleep apnea. Handb Exp Pharmacol. (2022) 274:181–201. doi: 10.1007/164_2021_558 34697666

[25] Kuvat N Tanriverdi H Armutcu F. The relationship between obstructive sleep apnea syndrome and obesity: a new perspective on the pathogenesis in terms of organ crosstalk. Clin Respir J. (2020) 14:595–604. doi: 10.1111/crj.13175 32112481

[26] Jordan AS McSharry DG Malhotra A. Adult obstructive sleep apnoea. Lancet. (2014) 383:736–47. doi: 10.1016/s0140-6736(13)60734-5, PMID: 23910433 PMC3909558

[27] Yuan G Nanduri J Khan S Semenza GL Prabhakar NR. Induction of HIF-1alpha expression by intermittent hypoxia: involvement of NADPH oxidase, Ca2+ signaling, prolyl hydroxylases, and mTOR. J Cell Physiol. (2008) 217:674–85. doi: 10.1002/jcp.21537 18651560 PMC2696817

[28] Chen LD Wu RH Huang YZ Chen MX Zeng AM Zhuo GF. The role of ferroptosis in chronic intermittent hypoxia-induced liver injury in rats. Sleep Breath. (2020) 24:1767–73. doi: 10.1007/s11325-020-02091-4, PMID: 32361960

[29] Young T Palta M Dempsey J Skatrud J Weber S Badr S. The occurrence of sleep-disordered breathing among middle-aged adults. N Engl J Med. (1993) 328:1230–5. doi: 10.1056/nejm199304293281704 8464434

[30] Young T Peppard PE Taheri S. Excess weight and sleep-disordered breathing. J Appl Physiol. (2005) 99:1592–9. doi: 10.1152/japplphysiol.00587.2005 16160020

[31] Jenkinson C Davies RJ Mullins R Stradling JR. Comparison of therapeutic and subtherapeutic nasal continuous positive airway pressure for obstructive sleep apnoea: a randomised prospective parallel trial. Lancet. (1999) 353:2100–5. doi: 10.1016/s0140-6736(98)10532-9 10382693

[32] Montserrat JM Ferrer M Hernandez L Farré R Vilagut G Navajas D. Effectiveness of CPAP treatment in daytime function in sleep apnea syndrome: a randomized controlled study with an optimized placebo. Am J Respir Crit Care Med. (2001) 164:608–13. doi: 10.1164/ajrccm.164.4.2006034 11520724

[33] Robinson GV Pepperell JC Segal HC Davies RJ Stradling JR. Circulating cardiovascular risk factors in obstructive sleep apnoea: data from randomised controlled trials. Thorax. (2004) 59:777–82. doi: 10.1136/thx.2003.018739, PMID: 15333855 PMC1747125

[34] Yokoe T Minoguchi K Matsuo H Oda N Minoguchi H Yoshino G. Elevated levels of C-reactive protein and interleukin-6 in patients with obstructive sleep apnea syndrome are decreased by nasal continuous positive airway pressure. Circulation. (2003) 107:1129–34. doi: 10.1161/01.cir.0000052627.99976.18, PMID: 12615790

[35] Pepperell JC Ramdassingh-Dow S Crosthwaite N Mullins R Jenkinson C Stradling JR. Ambulatory blood pressure after therapeutic and subtherapeutic nasal continuous positive airway pressure for obstructive sleep apnoea: a randomised parallel trial. Lancet. (2002) 359:204–10. doi: 10.1016/s0140-6736(02)07445-7, PMID: 11812555

[36] Pan YY Deng Y Xie S Wang ZH Wang Y Ren J. Altered Wnt signaling pathway in cognitive impairment caused by chronic intermittent hypoxia: focus on glycogen synthase kinase-3β and β-catenin. Chin Med J. (2016) 129:838–45. doi: 10.4103/0366-6999.178969 26996481 PMC4819306

[37] Wang Y Hai B Niu X Ai L Cao Y Li R. Chronic intermittent hypoxia disturbs insulin secretion and causes pancreatic injury via the MAPK signaling pathway. Biochem Cell Biol. (2017) 95:415–20. doi: 10.1139/bcb-2016-0167 28177762

[38] Madreiter-Sokolowski CT Thomas C Ristow M. Interrelation between ROS and ca (2+) in aging and age-related diseases. Redox Biol. (2020) 36:101678. doi: 10.1016/j.redox.2020.101678, PMID: 32810740 PMC7451758

[39] Chen J Zhu H Chen Q Yang Y Chen M Huang J. The role of ferroptosis in chronic intermittent hypoxia-induced lung injury. BMC Pulm Med. (2022) 22:488. doi: 10.1186/s12890-022-02262-x, PMID: 36572881 PMC9793575

[40] Gleitze S Paula-Lima A Núñez MT Hidalgo C. The calcium-iron connection in ferroptosis-mediated neuronal death. Free Radic Biol Med. (2021) 175:28–41. doi: 10.1016/j.freeradbiomed.2021.08.231, PMID: 34461261

[41] Ko J Jang S Kwon W Kim SY. Protective effect of GIP against monosodium glutamate-induced Ferroptosis in mouse hippocampal HT-22 cells through the MAPK signaling pathway. Antioxidants. (2022) 11:189. doi: 10.3390/antiox11020189, PMID: 35204073 PMC8868324

[42] Wu ZH Tang Y Yu H Li HD. The role of ferroptosis in breast cancer patients: a comprehensive analysis. Cell Death Dis. (2021) 7:93. doi: 10.1038/s41420-021-00473-5 PMC8097021 33947836

[43] Lee H Jiang X Perwaiz I Yu P Wang J Wang Y. Dopamine D(5) receptor-mediated decreases in mitochondrial reactive oxygen species production are cAMP and autophagy dependent. Hypertens Res. (2021) 44:628–41. doi: 10.1038/s41440-021-00646-w 33820956 PMC8369611

[44] Huang HX Yang G Yang Y Yan J Tang XY Pan Q. TFAP2A is a novel regulator that modulates ferroptosis in gallbladder carcinoma cells via the Nrf2 signalling axis. Eur Rev Med Pharmacol Sci. (2020) 24:4745–55. doi: 10.26355/eurrev_202005_21163, PMID: 32432738

[45] Wu L Wang W. Identification of hub genes in patients with Alzheimer disease and obstructive sleep apnea syndrome using integrated bioinformatics analysis. Int J Gen Med. (2021) 14:9491–502. doi: 10.2147/ijgm.s341078 34916831 PMC8668230

[46] Hoffmann MS Singh P Wolk R Narkiewicz K Somers VK. Obstructive sleep apnea and intermittent hypoxia increase expression of dual specificity phosphatase 1. Atherosclerosis. (2013) 231:378–83. doi: 10.1016/j.atherosclerosis.2013.09.033, PMID: 24267255 PMC3865929

[47] Wang X Tian Y Li C Chen M. Exploring the key ferroptosis-related gene in the peripheral blood of patients with Alzheimer's disease and its clinical significance. Front Aging Neurosci. (2022) 14:970796. doi: 10.3389/fnagi.2022.970796, PMID: 36118694 PMC9475071

