# Supplementary figures and images for: Ferroptosis-related genes are considered as potential targets for CPAP treatment of obstructive sleep apnea
Source: Front Neurol. 2023 Dec 21;14:1320954. doi: 10.3389/fneur.2023.1320954 (PMC10764456; doi:10.3389/fneur.2023.1320954)

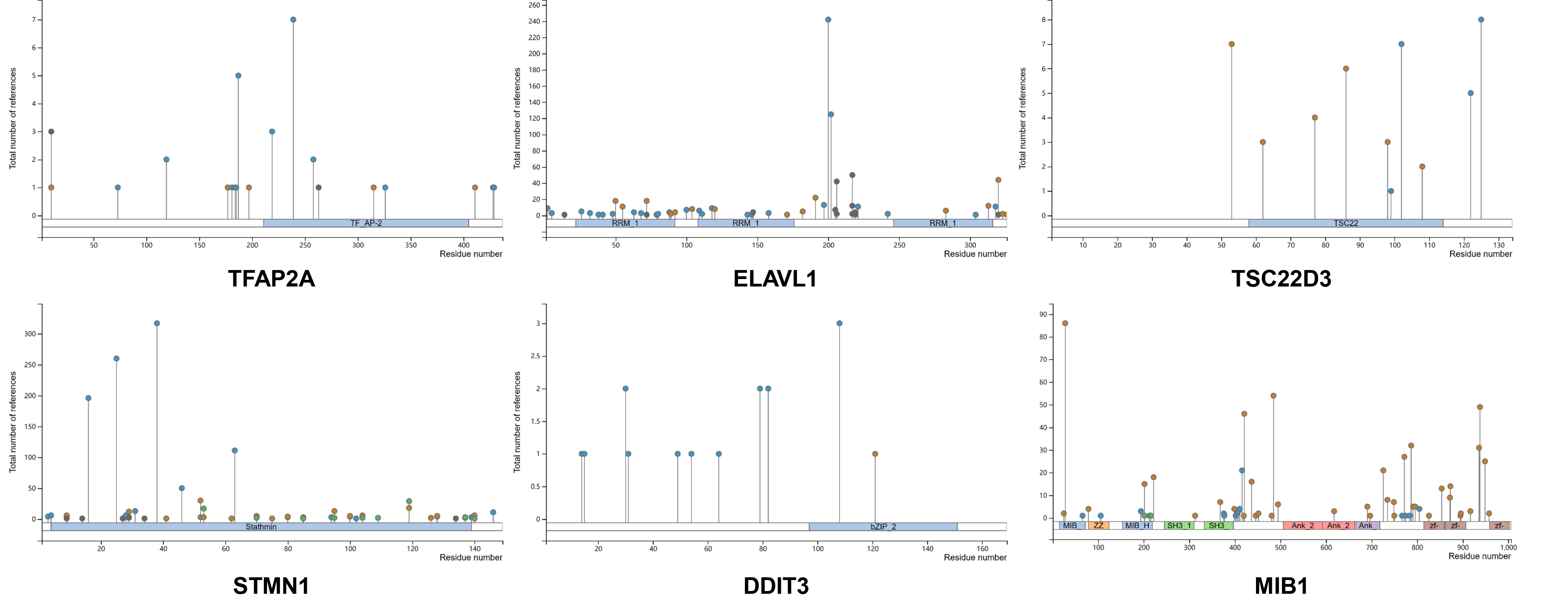

Supplement: SUPPLEMENTARY FIGURE S1 — PhosphoSitePlus lollipop plots of 6 ferroptosis-related genes. Circles 362 indicate PTM sites with a height reflecting the number of references describing the 363 site. [file Image_1.TIF]

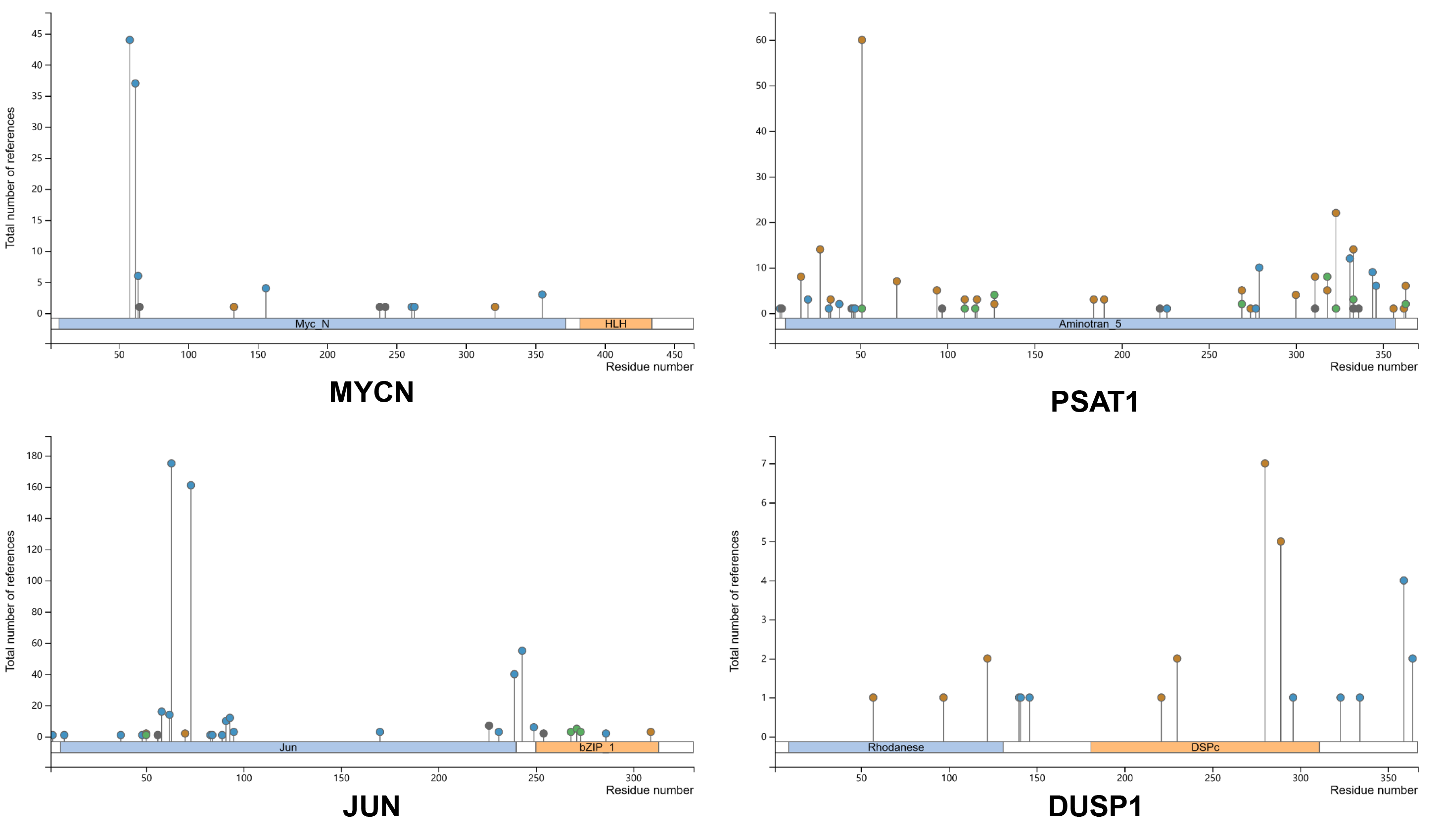

Supplement: SUPPLEMENTARY FIGURE S2 — PhosphoSitePlus lollipop plots of 4 ferroptosis-related genes. Circles indicate PTM sites with a height reflecting the number of references describing the site. [file Image_2.TIF]

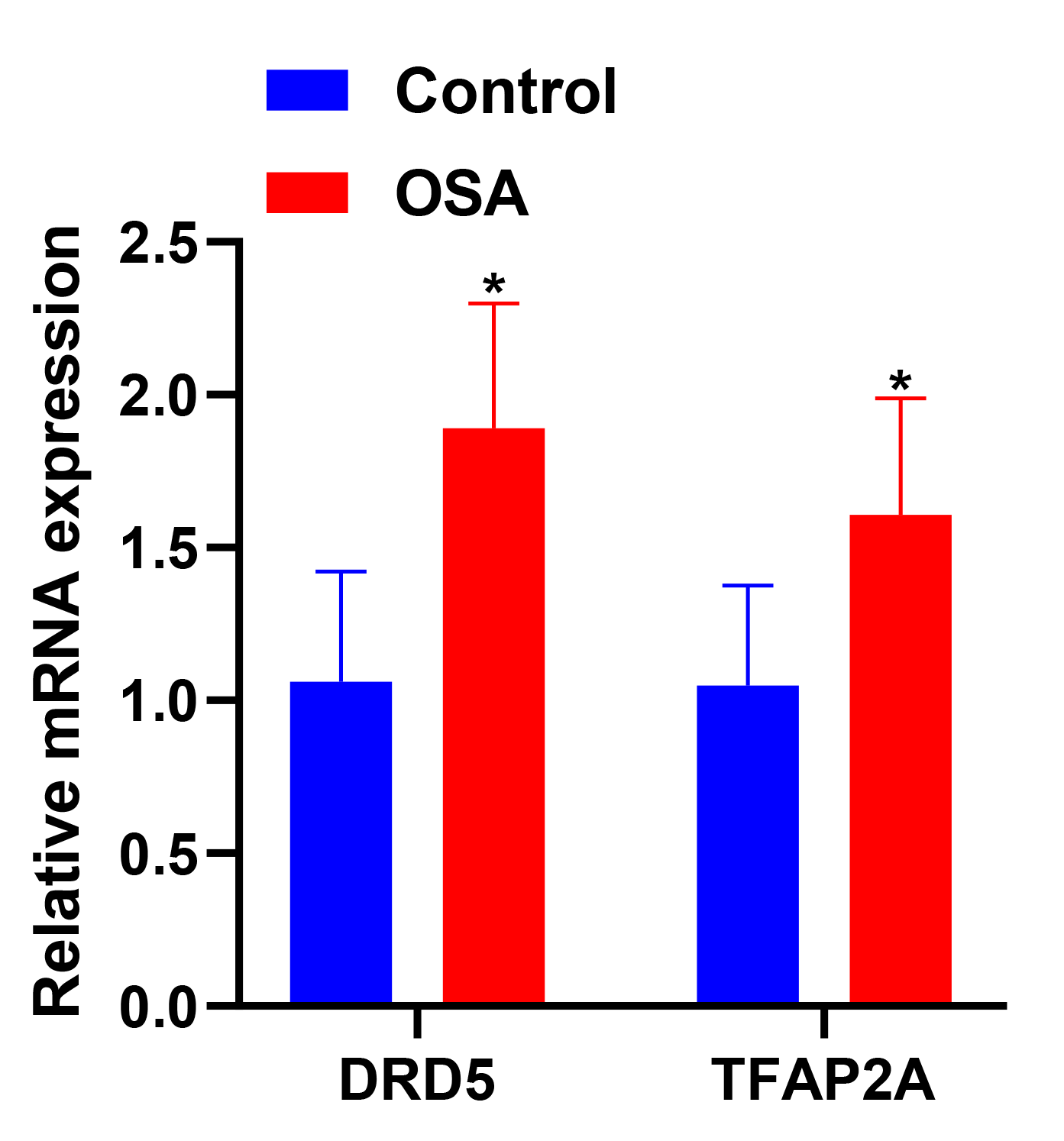

Supplement: SUPPLEMENTARY FIGURE S3 — Clinical validation of DRD5 and TFAP2A expression (*P < 0.05, n = 3). [file Image_3.TIF]
